# Oncogenic human papillomavirus-associated nasopharyngeal carcinoma: an observational study of correlation with ethnicity, histological subtype and outcome in a UK population

**DOI:** 10.1186/1750-9378-8-30

**Published:** 2013-08-12

**Authors:** Max Robinson, Yae-eun Suh, Vinidh Paleri, Debbie Devlin, Bushra Ayaz, Laura Pertl, Selvam Thavaraj

**Affiliations:** 1 Centre for Oral Health Research, School of Dental Sciences, Newcastle University, Newcastle Upon Tyne, UK; 2 Head and Neck Oncology, Guy’s and St. Thomas’ NHS Foundation Trust, London, UK; 3 Otolaryngology-Head and Neck Surgery, Newcastle upon Tyne Hospitals NHS Trust, Newcastle upon Tyne, UK; 4 Clinical and Diagnostic Sciences, King’s College London Dental Institute, London, UK

**Keywords:** Nasopharyngeal carcinoma, Human papillomavirus, Epstein-Barr virus

## Abstract

**Background:**

Nasopharyngeal carcinoma (NPC) accounts for 0.6% of all cancers worldwide with the highest prevalence in South East Asia, Southern China and Northern Africa but the disease is uncommon in Europe with an annual incidence in this region of less than 1 per 100 000. Although the Epstein-Barr virus (EBV) is a well known causative agent in NPC, recent reports have implicated oncogenic Human Papillomavirus (HPV) in a subgroup of these tumours. The recent striking rise of oropharyngeal carcinoma has been attributed to HPV, but little is known about the prevalence and clinical significance of the virus in NPC. The aim of this study was to determine the prevalence of oncogenic HPV in NPC from tissue archives of two head and neck cancer centres in the UK.

**Methods:**

Samples were available for 67 patients with clinically validated NPC. The detection of high-risk HPV was carried out by screening all cases for p16 using immunohistochemistry and HPV DNA by polymerase chain reaction (PCR) using GP5+/6+ primers. All cases with p16 over-expression or positive for HPV by PCR were then examined by high-risk HPV DNA in-situ hybridisation and genotype analysis by PCR.

**Results:**

Eleven cases (11/67, 16.4%) showed concurrent over-expression of p16 and evidence of high-risk HPV DNA by in-situ hybridisation; the majority were HPV16 positive. Of these 11 cases, nine occurred in Whites and two in Blacks. Histologically, there were two keratinising squamous cell carcinoma and nine non-keratinising carcinomas (eight differentiated and one undifferentiated). None of the HPV-positive cases showed any co-infection with EBV. There was no statistically significant difference in overall survival outcome between patients with HPV-positive and HPV-negative NPC.

**Conclusion:**

The results of this study show that oncogenic HPV is associated with a subgroup of NPCs and is more likely to occur in Whites. However, unlike oropharyngeal carcinoma there was no significant difference in overall survival between patients with HPV-positive and HPV-negative NPC.

## Background

Nasopharyngeal carcinoma (NPC) constitutes 0.6% of all cancers worldwide but there is a wide geographic and ethnic variation in the incidence rates of the disease [1]. For example, incidence rates of up to 30 per 100,000 have been reported in certain parts of China in contrast to <1 per 100,000 in North America [2,3]. In the United Kingdom, NPC is rare with a rate of 0.3-0.4 per 100,000 resulting in approximately 208 new cases per year [4]. The World Health Organisation (WHO) has classified NPC according to histological criteria into keratinising squamous cell carcinoma (KSCC, formerly WHO type I), non-keratinising carcinoma differentiated type (NK-D, formerly WHO type II), non-keratinising carcinoma undifferentiated (NK-U, formerly WHO type III) and basaloid squamous cell carcinoma (BSCC) [5]. The geographic and ethnic variation of this disease is also reflected in the histological subtype where KSCCs comprise around 25% and 2% of NPCs in North America and Southern China, respectively [6]. This disparity may be explained by differences in genetic and environmental factors.

Although the Epstein-Barr virus (EBV) has long been implicated in nasopharyngeal carcinogenesis, increasing numbers of reports have identified an association of oncogenic human papillomavirus (HPV) in a sub-group of NPCs [7-16]. Whilst HPV is now recognised as the causative factor for the recent striking increase in squamous cell carcinoma of the oropharynx in Europe and North America, similar epidemiological trends have not been observed in NPC [17]. Furthermore, little is known about the prevalence of oncogenic HPV in NPC and its clinical significance.

Therefore, the aim of this study was to determine the incidence of oncogenic HPV in patients with NPC from two head and neck cancer centres in the UK and to correlate HPV status with patient ethnicity, histological grade, EBV status and overall survival.

## Results

### Histological subtype

Of the 67 cases included in this study, there were 6 (8.9%) keratinising squamous cell carcinomas (KSCCs), 20 (29.9%) non-keratinising carcinomas, differentiated type (NK-D), 40 (59.7%) non-keratinising carcinomas, undifferentiated type (NK-U), and 1 (1.5%) was a basaloid squamous cell carcinoma (BSCC, Table 1). There was a statistically significant correlation between ethnicity (Far Eastern Asians, *p*=0.01 Chi square) and histological subtype (NK-U, *p* <0.001 Chi square).

**Table 1:** Summary of patient and tumour characteristics of all NPC cases

**Patient characteristic**	**Number (%)**
Gender	
Male	49 (73.1)
Female	18 (26.9)
Age mean, range	48, 12–74
Stage	
II	10 (14.9)
III	21 (31.3)
IV	35 (52.2)
Not available	1
Virus status	
EBV-HPV-	9 (13.4)
EBV+HPV-	47 (70.2)
EBV-HPV+	11 (16.4)
EBV+HPV+	0
Ethnicity	
White	34 (50.7)
Far Eastern Asian	17 (25.4)
Black	14 (20.9)
North African	2 (3.0)
Histological subtype	
KSCC	6 (8.9)
NK-D	20 (29.9)
NK-U	40 (59.7)
BSCC	1 (1.5)

### EBV status

Forty-seven (70.1%) were positive for EBV. Of these 17 (36.2%), 16 (34.0%), 12 (25.3%) and 2 (4.2%) occurred in Whites, Far Eastern Asians, Blacks and North Africans, respectively. One (2.1%) was a KSCC, 36 (76.6%) were NK-Us, 9 (19.2%) were NK-Ds, and 1 (2.1%) was a BSCC.

### HPV status

**Figure 1:**
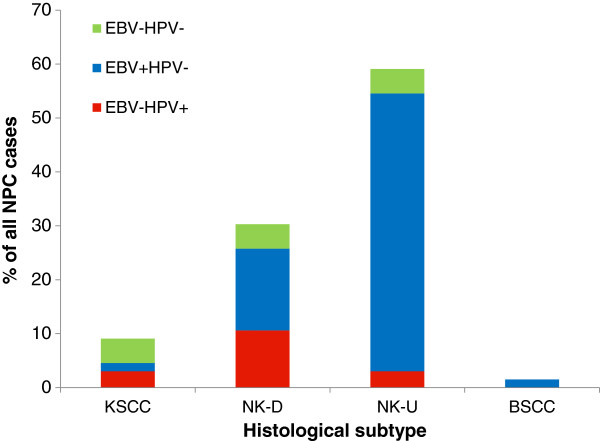
Viral status of NPCs by histological subtype of keratinising squamous cell carcinoma (KSCC), non-keratinising carcinoma, differentiated type (NK-D), non-keratinising carcinoma, undifferentiated type (NK-U) and basaloid squamous cell carcinoma (BSCC), expressed as percentage.

**Figure 2:**
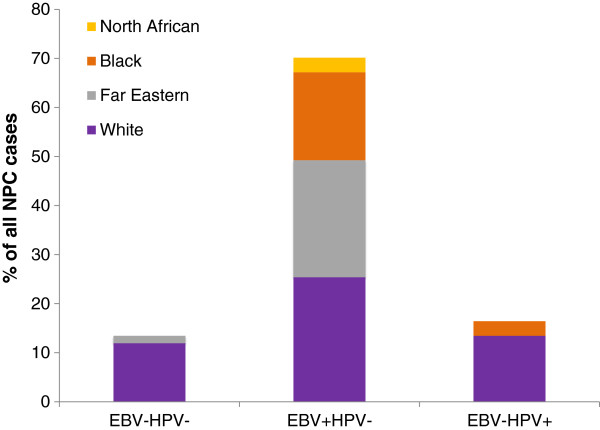
Viral status of all NPC cases subdivided by ethnicity, expressed as percentage.

Eleven (16.4%) cases demonstrated p16 over-expression, all of which were assigned a maximum H score of 300 (staining intensity × percentage tumour staining; 3×100=300) [18]. All cases over-expressing p16 were also positive for HPV DNA by consensus PCR and ISH for high-risk types. Among the 56 p16-negative cases, 51 had sufficient FFPE tissue for DNA extraction and amplifiable DNA was present in 40 of 51 samples. Two of these 40 cases were positive for HPV DNA by PCR, but were subsequently negative for high-risk HPV ISH. Of the HPV-positive NPCs, all were negative for EBV; 2/11 (18.2%) were KSCC, 7/11 (63.6%) were NK-D and 2/11 (18.2%) were NK-U (Table 2, Figure 1). 9/11 (81.8%) and 2/11 (18.2%) of HPV positive NPCs occurred in Whites and Blacks, respectively (Table 2, Figure 2). There were no HPV positive NPCs in Far-eastern Asians (Table 2, Figure 2). A statistically significant inverse correlation was seen between HPV and EBV status and Far-Eastern Asian ethnicity (both *p*<0.0001, Chi square). HPV-positive NPCs were more likely to occur in Whites (*p*=0.03, Chi square). Of the HPV-positive NPCs, 9/11 (81.8%) were genotyped as HPV16, 1/11 (9.1%) was HPV18 and there was insufficient amplifiable DNA in the remaining case.

**Table 2:** Summary of patient ethnicity and histological subtype with viral status

	**Viral status**			
	**Number (%)**			
	**EBV-/HPV-**	**EBV+/HPV-**	**EBV-/HPV+**	**EBV-/HPV-**
	***n=*9**	***n=*47**	***n=*11**	***n=*0**
Ethnicity				
White	8 (88.9)	17 (36.2)	9 (81.8)	0 (0)
Far-Eastern Asian	1 (11.1)	16 (34.0)	0 (0)	0 (0)
Black	0 (0)	12 (25.5)	2 (18.2)	0 (0)
North African	0 (0)	2 (4.3)	0 (0)	0 (0)
Histological subtype				
KSCC	3(33.3)	1 (2.1)	2 (18.2)	0 (0)
NK-D	4 (44.4)	9 (19.2)	7 (63.6)	0 (0)
NK-U	2 (22.2)	36 (76.6)	2 (18.2)	0 (0)
BSCC	0 (0)	1 (2.1)	0 (0)	0 (0)

### Overall survival

**Figure 3:**
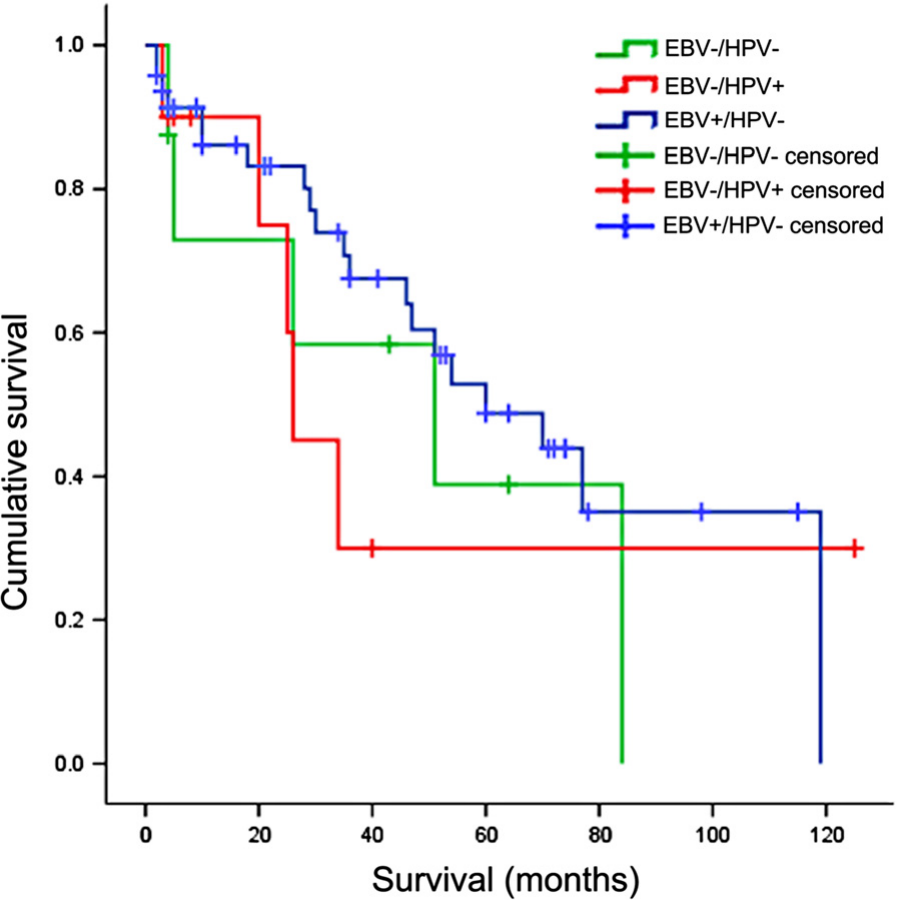
**Kaplan-Meier plot for overall survival of NPC by viral status (EBV-/HPV-, EBV+/HPV- and EBV-/HPV+).** There was no statistically significant difference in the overall survival between these groups (p=0.57, Log Rank).

Patients were followed up for a period of 4–120 months (mean 33.7, median 26.0). The mean overall survival for all cases was 63.1 months (95% CI 49.6-76.5) whereas the mean overall survival for EBV-negative/HPV-negative, EBV-positive/HPV-negative and EBV-negative/HPV-positive were 47.6 months (95% CI 19.9-75.3), 67.9 months (95% CI 52.1-83.7) and 53.6 months (95% CI 18.3-88.8), respectively. There was no statistically significant difference in the mean overall survival between EBV-negative/HPV-negative, EBV-positive/HPV-negative and EBV-negative/HPV-positive patients (*p*=0.57, log rank; Figure 3).

## Discussion

Oncogenic HPV is now recognised as the causative factor for the striking increase in the incidence of cancers of the oropharynx in recent decades in North America and Western Europe. This phenomenon is accompanied by the observation that patients with HPV-related oropharyngeal cancer have a significantly improved overall- and disease-specific survival compared to site-matched HPV-negative cancers. Although HPV-related head and neck cancers have a strong site predilection for the oropharynx, the virus is also known to be associated with carcinomas in other head and neck sites including the nasopharynx [19]. However, while the biological behaviour of HPV-related oropharyngeal carcinoma is now becoming increasingly established, little is known about the clinical significance of this virus in NPC. This study therefore aimed to address this knowledge gap by determining the incidence of oncogenic HPV in NPC and to correlate viral status with patient ethnicity, histological grade and overall survival.

There are morphological and functional similarities between oro- and naso-pharyngeal mucosa. Both sites contain mucosa-associated lymphoid tissue with reticular crypt epithelia that are specialised for trans-epithelial antigen processing. These similarities, together with the close anatomical proximity between these two sites have led Singhi *et al.* to speculate that HPV-related carcinomas of the nasopharynx likely represent secondary extension from the oropharynx [14]. Furthermore, since all HPV-related nasopharyngeal tumours in their series demonstrated oropharyngeal continuity, they maintain confidence that detection of HPV in metastatic squamous cell carcinomas in cervical nodes should direct the search for the primary tumour to the oropharynx. By contrast, in the current study, all tumours were radiologically confirmed as centred in the nasopharynx without any evidence of continuity with oropharyngeal mucosa. The identification of HPV-related carcinomas of truly nasopharyngeal origin raises the necessity of including the nasopharyngeal mucosa in the search for the primary tumour in patients presenting with HPV-positive metastatic carcinomas from an unknown origin. Significantly, these data suggest that it may not be oncologically safe to exclude the nasopharynx from the field of therapeutic irradiation for these patients despite the greater levels of toxicity.

The reported incidence of HPV in NPC ranges from 9–52.9% [8-10,12-14]. Although this wide range may be attributed to differences in geographic variation with regard to aetiology and prevalence of NPC, it is more likely a result of non-standardised viral detection methods. HPV DNA detection by PCR alone is known to result in false positives [20,21] which explains why the incidence of the virus in NPC is much lower when surrogate markers for oncoprotein expression are used in combination with DNA in-situ hybridisation [14]. The current study employed a previously described algorithm that combines an initial screening using p16 immunohistochemistry, a highly sensitive surrogate marker for viral oncoprotein, followed by highly specific high-risk HPV DNA in-situ hybridisation [21]. This multi-tier approach is likely therefore to represent a more accurate assessment of the incidence of biologically significant oncogenic HPV in NPC.

An increasing body of evidence now indicates that patients with HPV-positive oropharyngeal squamous cell carcinoma have a better overall- and disease-specific survival compared to site-matched HPV-negative patients [17]. However, little is known about the prognostic significance HPV detection in non-oropharyngeal head and neck cancer including those that arise within the nasopharynx. Since, to our knowledge, only a single report has evaluated the clinical significance of HPV in this disease, we sought to correlate viral status with overall survival in our cohort of NPC patients [16]. We found no significant difference in overall survival between patients with HPV-positive and HPV-negative NPCs. Therefore, unlike HPV-associated oropharyngeal carcinoma, the oncogenic papillomavirus status in NPC is unlikely to influence survival outcome. These findings are timely since there are now calls for treatment de-escalation for patients with HPV-positive oropharyngeal carcinoma based on improved survival outcome. Our data suggest that improved survival in HPV-positive tumours is likely to be limited to the oropharyngeal carcinomas and there is currently no evidence to support any changes in NPC treatment protocols based on oncogenic HPV status. However, larger cohort studies are necessary to confirm the lack of difference in survival outcome between HPV-positive and HPV-negative NPC.

Evidence from UK-based studies indicates that 57-61% of oropharyngeal carcinomas are attributable to high-risk HPV [21,22]. Whilst data is emerging for the incidence rates of HPV-associated cancer in non-oropharyngeal head and neck sites, the current study is the first to provide such data in NPC within an ethnically diverse UK population [23]. The overall incidence of 16.4%, is in keeping with other studies from Western centres using similar detection methods [12,14]. Although these tumours are rare in this population, our data suggest that NPC should be included when evaluating the overall burden of HPV-associated disease.

Our findings also confirm previous reports that HPV-associated NPC is more likely, but not exclusively, to occur in Whites [11]. The reason for the predilection of HPV in this group of patients is unknown. Interestingly, while some groups have identified HPV and EBV co-infection, we and others demonstrate an inverse relationship between these viruses such that all HPV-associated tumours do not harbour EBV [8,11]. Our data support current guidelines from the College of American Pathologists recommending HPV testing in EBV-negative NPCs [24]. Although viral status is unlikely to influence treatment decisions and outcome, an accurate incidence and prevalence of oncogenic HPV in NPC is important in order to ascertain the true disease burden of HPV-related malignancy.

## Conclusion

We confirm the presence of high-risk HPV in a subgroup of NPC patients. HPV-16 is the most commonly implicated genotype and prevalence of HPV-positive NPC is 16.4% in this UK population of mixed ethnicity. HPV-associated NPC is more likely to occur in Whites, but there is no association between histological grade and HPV status in these tumours. Unlike HPV-related oropharyngeal carcinoma, HPV status is not associated with improved overall survival outcome in NPC. Larger cohort studies are needed to confirm these findings and to characterise the biological and clinical significance of HPV in NPC.

## Methods

### Ethics

This study has had National Research Ethics Service review (12/SC/0151) and has been registered with National Health Service Research and Development (RJ112/N247).

### Patients and samples

Patients presenting to two UK head and neck cancer centres (Guy’s and St. Thomas’ NHS Foundation Trust, London and Newcastle-upon-Tyne Hospitals NHS Foundation Trust) were identified retrospectively from pathology databases over a period of 12 years (2000–2012). Only tumours radiologically confirmed as centred in the nasopharynx without extension into the oropharynx were included in this study. Formalin-fixed paraffin embedded (FFPE) tissue was available from 67 patients of which 48 were males and 19 females (M:F ratio 2.5:1) with a mean age of 48 years (range 12–74 years old). There were 34 Whites (50.7%), 17 Far-Eastern Asians (25.4%), 14 Blacks (20.9%), and two North Africans (3.0%). Patient and tumour characteristic are summarised in Table 1.

All cases were independently classified according to WHO histological criteria by two specialist head and neck pathologists (MR and ST) [5]. A consensus was reached for tumours with discordant grades. Ethnicity and patient survival were determined by review of patient treatment records.

### p16 immunohistochemistry

**Figure 4:**
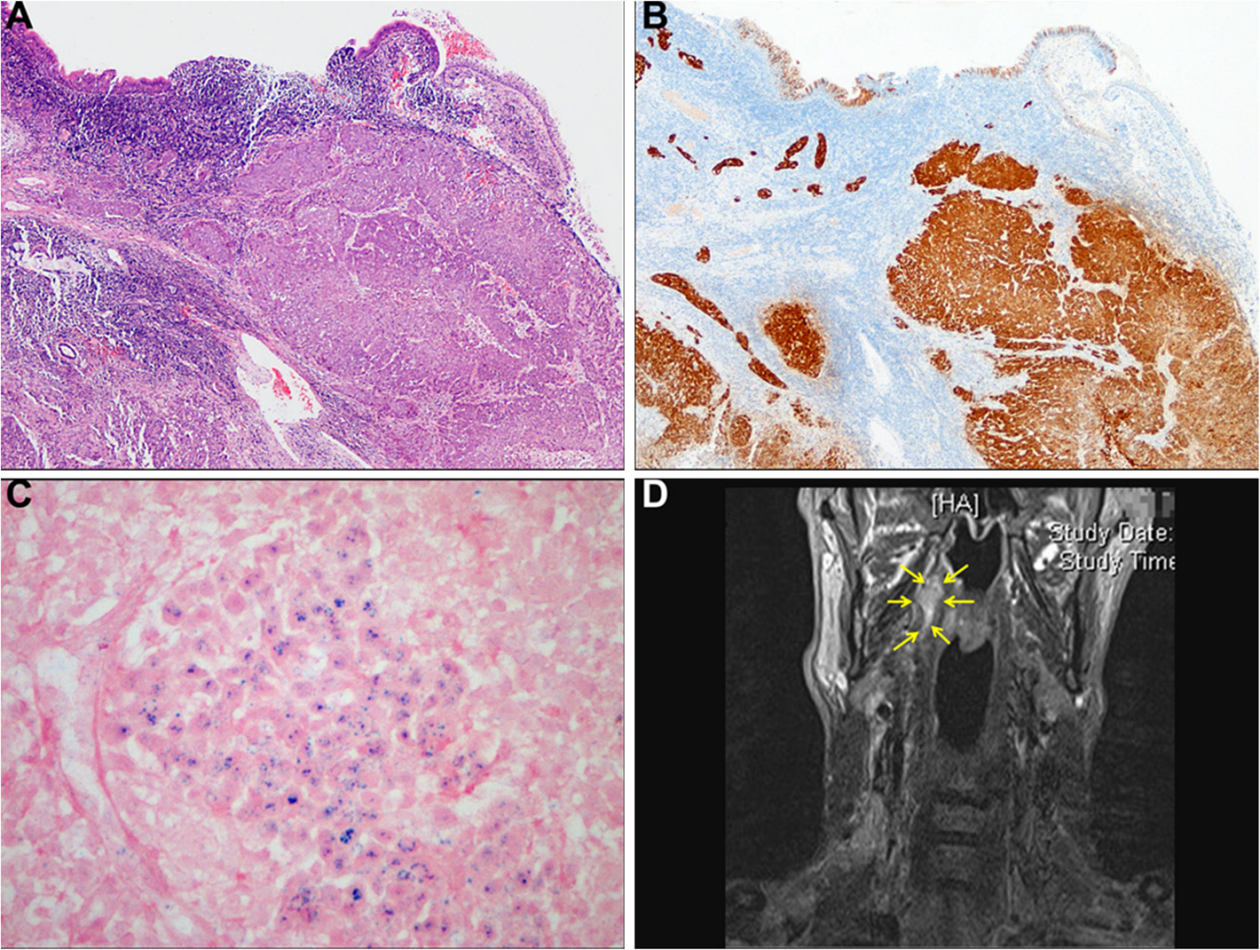
**An HPV-positive NPC. A**-**C**. Representative photomicrographs of H&E **(A)**, p16 IHC **(B)** and high-risk HPV DNA ISH **(C)**. **D**. Coronal section of T2 STIR MRI image of the same patient. Arrows delineate tumour in the right nasopharynx.

p16 immunohistochemistry (IHC) was performed on all cases using a proprietary kit (CINtec Histology, mtm Laboratories AG, Germany) on a Ventana Benchmark Autostainer (Ventana Medical Systems Inc. USA). A tonsil carcinoma with high p16 expression was used as a positive control. The primary antibody was omitted from negative controls. The tests were independently scored by two specialist head and neck pathologists (MR & ST) using a binary scoring system and the recently described H score (Figure 4) [18]. Discordant scores were resolved by consensus at a meeting between the pathologists.

### Polymerase chain reaction

DNA was extracted from 25μm sections of FFPE biopsies using the QIAamp DNA (FFPE) tissue kit (Qiagen, Crawley, UK) according to the manufacturer’s instructions. Contamination of samples with extrinsic DNA was minimised by cleaning the microtome with xylene and discarding the microtome blade between each case. PCR reactions were carried out using standard operating procedures to prevent sample contamination. All DNA samples were screened for the human β-globin gene by PCR to verify the presence of amplifiable DNA [25]. HPV DNA was analysed by PCR using the improved general primer set GP5+/6+ which amplifies part of the HPV L1 gene encoding the HPV major capsid protein, detecting low-risk genotypes −6, -11, -40, -42, -43 and −44 and high-risk genotypes −16, -18, -31,-33, -35, -39, -45, -51, -52, -56, -58, -59, -66 and −68 as previously described [26]. DNA from the UPCI: SCC089 and SCC090 oropharyngeal squamous cell carcinoma cell lines were used as positive and negative controls, respectively [27]. A binary classification of presence or absence of an amplicon of appropriate size on resolving gels was employed.

### In-situ hybridisation

EBV RNA in-situ hybridisastion (ISH) was performed using proprietary reagents (Inform EBER DNP RNA probe, Ventana Medical Systems Inc, UK) on a Benchmark Autostainer (Ventana Medical Systems Inc. UK) and visualised using the Ventana ISH/iView Blue detection kit according to manufacturer’s instructions. The Inform EBER probe detects early RNA transcripts EBER-1 and EBER-2. Cells known to contain high numbers of EBER transcripts were supplied by the manufacturer and used as a positive control. An intense blue reaction product in the majority of malignant cells was considered positive. The tests were independently scored by the study pathologists using a binary classification and a consensus agreement was reached for all discordant scores.

All cases over-expressing p16 were subject to high-risk HPV DNA ISH using proprietary reagents (Inform HPV III Family 16 Probe (B), Ventana Medical Systems Inc, USA) on a Benchmark Autostainer (Ventana Medical Systems Inc. UK). The Inform HPV III Family 16 Probe (B) detects high-risk genotypes HPV-16, -18, -31, -33, -35, -39, -45,-51, -52, -56, -58 and −66. Three control samples were used: FFPE CaSki cells (HPV-16-positive; 200–400 copies per cell), HeLa cells (HPV-18-positive; 10–50 copies per cell) and C-33A (HPV-negative; Ventana Medical Systems Inc. USA). The high-risk HPV ISH test was scored as positive if there was any blue reaction product that co-localised with cell nuclei. Diffuse nuclear and cytoplasmic (‘episomal’ pattern) staining and punctate nuclear (‘integrated’ pattern) staining were scored as positive (Figure 4). Focal specific staining of only part of the tumour section was regarded as positive. Pale staining limited to the nucleoli of cells and staining of occasional leucocytes and stromal cells were also disregarded, in line with the manufacturer’s instructions.

The tests were independently scored by the two study pathologists using a binary classification as previously described and a consensus agreement was reached for all discordant scores [21]. Only cases demonstrating p16, PCR and ISH positivity were considered as HPV-positive.

### HPV genotype analysis

DNA was extracted from 10μm curls of formalin-fixed paraffin embedded tissue using the Cobas DNA extraction kit (Roche Molecular Systems Inc. USA). DNA yield was measured using a Nanodrop spectrophotometer (Thermo Scientific, USA). 200ng of DNA was diluted in 1000 μl of Surepath fluid (BD Diagnostics-TriPath, USA) and the samples were analysed using the Cobas HPV test on the Cobas 4800 instrument (Roche Molecular Systems Inc. USA).

### Statistical analysis

The Chi-squared test was used to evaluate the correlation between different groups. Overall survival was defined as the time from diagnosis to the date of death or last follow up. The Kaplan-Meier method was used to estimate time to event curves, and the survival curves were compared using the log-rank test. *P* values below 0.05 were regarded as significant. All data was analysed using SPSS software version 20.

## Abbreviations

EBV: Epstein-Barr virus; HPV: Human papillomavirus; IHC: Immunohistochemistry; ISH: In-situ hybridisation; NPC: Nasopharyngeal carcinoma; PCR: Polymerase chain reaction.

## Competing interests

The authors declare that they have no competing interests.

## Authors’ contributions

MR was involved in the conception of the study, identified the cases, undertook histological evaluation, carried out genotype analysis, participated in the design and coordination and helped to draft the manuscript. YS undertook DNA extraction, data collection from treatment records and performed statistical analyses. VP undertook data collection and was involved in drafting and critical evaluation of the manuscript for intellectual content. DD performed immunohistochemistry and in-situ hybridisation assays. BA performed PCR assays. LP supported immunohistochemistry and in-situ hybridisation assays. ST was involved in the conception of the study, identified the cases, undertook histological evaluation, participated in the design and coordination and drafted the manuscript. All authors read and approved the final manuscript.
